# CD100 on NK Cells Enhance IFNγ Secretion and Killing of Target Cells Expressing CD72

**DOI:** 10.1371/journal.pone.0000818

**Published:** 2007-09-05

**Authors:** Sa'ar Mizrahi, Gal Markel, Angel Porgador, Yuri Bushkin, Ofer Mandelboim

**Affiliations:** 1 The Lautenberg Center for General and Tumor Immunology, Hebrew University-Hadassah Medical School, Jerusalem, Israel; 2 Department of Microbiology and Immunology, Faculty of Health Sciences, The Cancer Research Center, Ben Gurion University, Beer-Sheva, Israel; 3 Public Health Research Institute, NJMS-UMDNJ, Newark, New Jersey, United States of America; New York University School of Medicine, United States of America

## Abstract

**Background:**

NK cells are able to kill tumor and virus-infected cells without the need of prior antigen stimulation. The killing of these target cells is regulated by inhibitory, lysis and co-stimulatory receptors that are expressed on the surface of NK cells.

**Principal Findings:**

CD100 (Semaphorin 4D), a 150kD transmembrane protein, is expressed on the surface of activated NK cells as a homodimer, mediates the killing of target cells by binding to CD72. CD100 is not involved directly in the killing process but is rather increases NK cytotoxicity by enhancing the adhesion between NK cells and their targets. This increased adhesion leads to a more efficient killing and enhanced IFNγ secretion.

**Significance:**

Since CD72 is expressed on antigen presenting cells (APC) and the CD100-CD72 interaction lead to the shading of CD100, we suggest that NK interacting with APC cells could be the early source of soluble CD100 which is crucial for the formation of antigen specific immune response. CD100-CD72 interaction can be the mechanism by which NK cell communicate with B cells.

## Introduction

NK cells are part of the innate immunity system and are able to kill tumor and virus-infected cells that have lost, in most cases, class I MHC protein expression [1].The recognition of MHC class I proteins by NK inhibitory receptors leads to inhibition of NK killing and in the absence of MHC class I proteins, these inhibitory constraints are removed and NK cytotoxicity is restored[2]–[9]. In recent years it was realized however that NK cytotoxicity is much more complicated [10] and that the killing of NK cells is also controlled by activating receptors, among these are Natural Cytotoxicity Receptors (NCRs), NKG2D, CD16 (low affinity FcγRIII), 2B4 and NKp80 [11]. NK cells are also capable of producing cytokines, including TNFα, GM-CSF, and a large quantity of IFNγ. IFNγ affect many cellular responses, including the control of viral replication, up-regulation of MHC class I and class II protein expression and activation of macrophages. It can also direct the adaptive immune responses towards the Th1 type [12].

The semaphorins which are characterized by “Sema” domain (∼500 A.A.) in their extracellular region were initially recognized for their role as chemorepellents during neural development [13]. The semaphorin CD100 is the first semaphorin to be found on the surface of immune cells[14]–[16] and is the best semaphorin characterized so far [15], [17]–[20]. Membrane bound CD100 is a 150-kDa trans-membrane protein, express as a homodimer [14]–[16] with high levels of expression both in lymphoid organs such as thymus, spleen and lymph node, and on non- lymphoid organs such as brain, kidney and heart [14], [15], [21].On hematopoietic cells it can be found on resting T cells, B cell, macrophages, dendritic cells (DC) and its expression is up-regulated significantly after cellular activation [15], [16], [22], [23]. CD100 can be cleaved from the membrane to form a functional soluble homodimer in the size of 240-kDa [24]–[26]. Two distinct receptors were identified for CD100: plexin-B1, which is the high affinity receptor for CD100, is found on many tissues with high levels of expression in the fetal brain and kidney [27].The low affinity receptor for CD100 is CD72, the major receptor for CD100 in immune cells [22]. CD72 is expressed during all stages of B cell maturation, except for plasma cells [22], and is also expressed on other antigen presenting cells such as dendritic cells and macrophages [28], [29].

CD100 has many biological activities in the immune system. It enhances B cells response to stimulation with CD40 and LPS both in vitro and in vivo [15], [16], [22], [23], [30]. B cells derived from CD100-/- knockout mice demonstrate a reduction in B cell activity and antibodies specific to T cell dependent (TD) antigens [31]. In contrast transgenic mice expressing functional soluble CD100 demonstrate the reverse pattern [32].

CD100 has also been found to have an important function in DC. CD100-deficient mice were resistant to autoimmune diseases models such as experimental autoimmune encephalomyelitis (EAE) [28] and immune complex glomerulonephritis (ICG) [33]. This effect was due to the lack of proper mature DC in the CD100 knockout mice. In human monocytes, soluble CD100 inhibits migration and induce the production of pro-inflammatory cytokines [28] and inhibit their migration [26], [34]. Here, by screening for novel antibodies that affect NK killing we identified a new mAb that recognizes CD100. Using this mAb we demonstrate a novel role for CD100 in the augmentation of NK killing and cytokine secretion.

## Results

### Identification of 172.4 mAb which recognize ligand that is up-regulated after activation of NK cells

**Figure 1:**
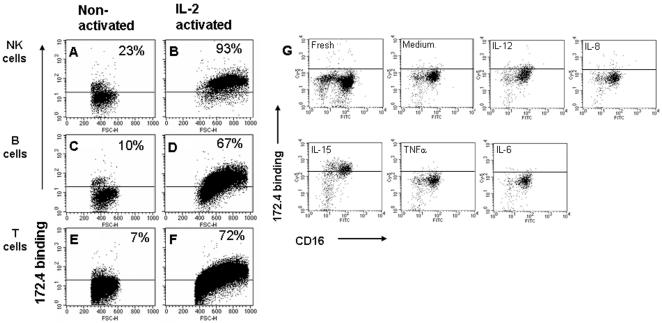
Expression of the ligand of 172.4 on lymphocyte sub-populations and NK line. Biotinylated 172.4 mAb was used in combination with other fluorescently labeled mAbs in a four color staining. 172.4 staining was detected using Cy-5 streptavidin. Cells were analyzed by flow cytometry. T cells were identified as CD3 positive, NK cells as CD56 positive, CD3 negative, and B cells as CD19 positive. Staining was performed on freshly isolated PBL (A, C and E) and on PBL that were cultured for 72 hr in the presence of IL-2 (100 u/ml) and human serum (B, D and F). Each dot blot shows a gated specific sub-population. An isotype matched antibody was used to determent the background staining for each antibody and is represented in the figure as the horizontal line. (H) Freshly isolated PBMC were incubated for 72 hr with 50 ng/ml of the indicated cytokines. Biotinylated 172.4 mAb was detected using Cy-5 streptavidin and used in combination with other fluorescently labeled mAbs in a four color staining. The PBL were analyzed for the expression of CD100 on NK cells and each dot plot represents a gated NK cells (CD3-, CD56+). Figure shows one representative experiment out of four performed.

Several NK receptors such as NKp44 on NK [35] and NKG2D on T cells [36] are upregulated after activation. Based on that fact, our assumption was that the level of other yet unknown NK activating receptors is also up-regulated after NK cell activation. In order to identify such receptors we immunized mice with the NK cell line-YTS. 2000 hybriomas were obtained and the supernatants of these hybridomas were tested for the binding to IL-2 activated and non-activated peripheral blood lymphocytes (PBL) by flow cytometry. Increased binding to IL-2 activated PBL compared to fresh PBL was observed with the supernatant of hybridoma 172 (data not shown). Hybridoma 172 was re-cloned and a mAb was purified from the hybridoma sub line named 172.4. Supernatants from 172.4 were tested in binding to different sub-population of activated and non-activated PBL. As shown in Figure 1, 172.4 mAb recognizes a sub-population of resting NK, B and T cells (figure 1 A, C, E). A dramatic increase in the binding of 172.4 to all tested populations was observed after 72 hr of activation with IL-2 (figure 1 B, D and F). Almost all activated NK cells express high levels of the ligand for 172.4 (93% in figure 1B).

To further characterize the 172.4 expression on NK cells, we activated the NK cells with different stimuli. The two main NK populations in the blood are CD16^−^, CD56^bright^ and the CD16^+^, CD56^dim^. They differ both in function and in expression of different molecules on their surface [37], [38]. Fresh PBMC were incubated with different stimulates (50 ng/ml) for 72 hr and the expression of 172.4-ligand on NK cells was analyze by flow cytometry. As can be seen in figure 1G enhancement of 172.4-ligand expression was observed only after incubation with IL-12 and more pronouncedly after incubation with IL-15. Both cytokines are well known activators of NK cells [39], [40]. In contrast, incubation with IL-8, IL-6 and TNFα had no effect. IL-12 stimulation of NK cells resulted in modest elevation of the 172.4-ligand on CD16^+^ NK cells (median fluorescence intensity (MFI) 90.2 verses 60.4 for the negative control) but not on CD16^−^ cells (MFI of 59.9 verses 53.3 for the negative control). IL-15 treatment resulted in marked 172.4-ligand expression on both populations (MFI of 235 and 269 in CD16^−^ and CD16^+^, respectively).

Thus, the protein recognize by 172.4 mAb is elevated in a response to a variety of activation stimuli which include IL-12, IL-15 and IL-2 and therefore can serve as a marker of NK activation.

### The ligand recognized by 172.4 mAb is not directly involved in the killing of target cells by NK cells

**Figure 2:**
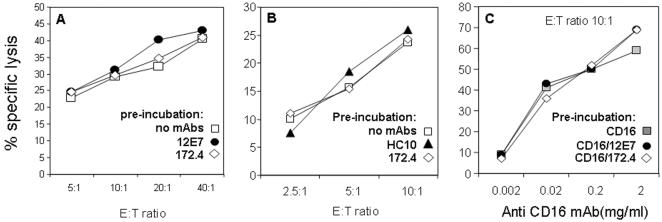
Killing of target cells is not influence by mAb 172.4. (A) ^35^S-labeled 721.221 cells were pre-incubated with the indicated mAbs and tested for lysis by NK cells at different effectors to targets (E:T) ratios as indicated in the figure. (B) ^35^S-labeled P815 cells were pre-incubated with the indicated mAbs and tested for lysis by NK cells at the indicated E:T ratios. (C) ^35^S-labeled P815 cells were pre-incubated with the combination of the indicated mAbs and tested for lysis by NK cells. E: T ratio of 10∶1 is presented. Figure shows one representative experiment out of four performed.

To test whether the protein recognized by 172.4 mAb is involved in the regulation of NK cytotoxicity we performed lysis assays. IL-2-activated NK cells were pre-incubated with various mAbs, indicated in figure 2A, and then subjected to lysis assay with ^35^S-labeled 721.221 or RPMI 8866 as target cells. No differences in the lysis of 721.221 (figure 2A) or RPMI 8866 (data not shown) were observed regardless of whether the control 12E7 mAb or 172.4 mAb were included in the assay. We concluded that the protein recognized by mAb 172.4 is not directly involved in killing of 721.221 or RPMI 8866 cells. However, it is still possible that the protein recognized by mAb 172.4 is involved in the killing of other cells by NK cells. We, therefore, tested whether this protein is involved in the regulation of NK cell-mediated cytotoxicity by performing re-directed lysis experiments (figure 2B and 2C). ^35^S-labeled P815 cells were incubated with different mAbs, as indicated in figure 2B, and then incubated with NK cells. As above, no differences were observed when either the control mAb HC10 or mAb 172.4 were included in the assay. Thus the protein recognized by172.4 is not directly involved in NK cytotoxicity. Next, we tested whether the protein recognized by mAb 172.4 may act as co-stimulatory molecule on NK cells. P815 cells were pre-incubated with increasing concentrations of the anti-CD16 mAb B73.1.1 combined either with mAb 172.4 or control mAb 12E7, and then incubated with the NK cells. Efficient lysis of P815 cells was observed even when cells were incubated with 0.02 µg/ml of B73.1.1 mAb (figure 2C). As above, no differences in lysis of P815 cell were observed when either mAb 12E7 or 172.4 mAb were included in the assay together with B73.1.1. Thus, the protein recognizes by mAb 172.4 is unlikely to be directly involved in the regulation of NK cell cytotoxicity.

### The protein recognized by mAb 172.4 is CD100

**Figure 3:**
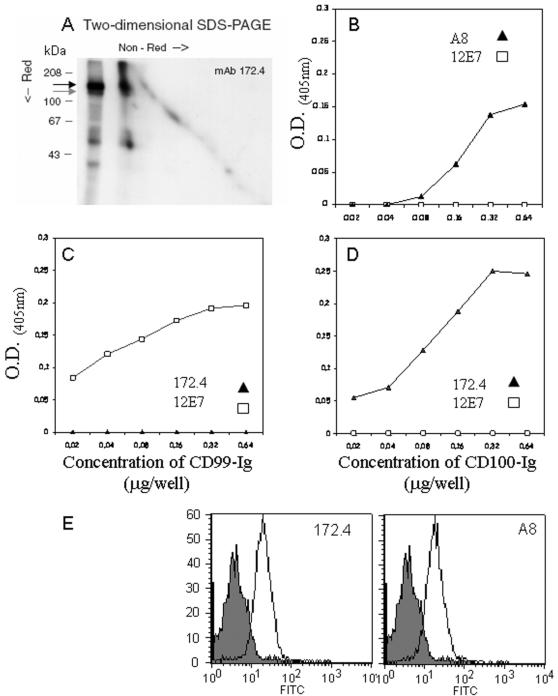
172.4 recognize CD100, a 150kDa protein expressed as a homodimer. (A) Surface radioiodinated YTS cell lysate was immunoprecipitated with mAb 172.4. The immunoprecipitate was analyzed first on non-reducing and then on reducing SDS-PAGE. The left lane is an aliquot of immunoprecipitated proteins resolved under reducing conditions. Molecular weight markers are as shown. The two forms of CD100 (150 and 120 kDa respectively) are indicated with arrows. (B, D) ELISA plates were coated with CD100-Ig and assayed for binding to the indicated antibodies as described in “Materials and Methods”. (C) ELISA plates were coated with CD99-Ig and assayed for binding to the indicated antibodies as described in “Materials and Methods”. The background binding to BSA was subtracted. Figure shows one representative experiment out of four performed. (E) IL-2 activated NK line was stained with either 172.4 or the commercial anti human CD100 antibody A8. FITC conjugated Goat F(ab') anti mouse IgG antibody was used as secondary Ab. Gray histogram is staining with secondary antibody only. Figure show one representative experiment out of 5 performed.

To identify the protein recognized 172.4, we immunoprecipitated the protein with mAb 172.4 from lysate of ^125^I-labeled YTS cells and analyzed it by two-dimensional (non-reducing/reducing) SDS-PAGE gel. As shown in figure 3A, mAb 172.4 immunoprecipitated a major protein of ∼150kDa under reducing conditions. This protein runs as a broad band of about 150–300kDa under non-reducing conditions (data not shown), thus implying that the 150kDa protein recognized by mAb 172.4 could form homodimers (figure 3A). The minor ∼120kDa protein band, under reducing condition, that was also precipitated by mAb 172.4 might be a proteolyticly cleaved fragment (while the other minor 53kDa protein band is non-specific). A data bank search suggested that the protein recognize by 172.4 might be CD100. CD100 is a 150kDa protein expressed as 300kDa homodimer protein on the surface of T cells and B cells [14], [16], [25], [41]. A soluble form, a homodimer composed of two sub-units of 120kDa each has also been described [25], [42].

To test whether the 172.4 mAb recognizes CD100 we first performed flow cytometry analysis of various cell types, using both 172.4 and the commercial anti-CD100 mAb A8 and observed identical staining patterns (data not shown). To prove that 172.4 recognize CD100 we used a fusion protein, composed of the extracellular region of human CD100 fused to Fc region of human IgG1 (CD100-Ig). We produced the CD100-Ig protein in COS-7 cells as previously described [43]–[45] and performed ELISA with increasing concentrations of CD100-Ig and CD99-Ig (used as control) fusion proteins. Figure 3 shows that both the commercial anti-CD100 mAbs A8 (figure 3B) and 172.4 (figure 3D) recognized CD100-Ig but not CD99-Ig, whereas 12E7 recognized only the CD99-Ig (figure 3C).

To further verify that mAb 172.4 recognize human CD100 we compare the staining of human NK line by 172.4 mAb to that of the commercial mAb A8 (figure 3E). All activated NK cells were stained positive for both antibodies and demonstrated an identical pattern of staining for both antibodies.

We therefore concluded that mAb 172.4 recognize the protein known as CD100.

### CD100 on NK cells enhance the killing and IFNγ secretion after incubation with targets expressing CD72

**Figure 4:**
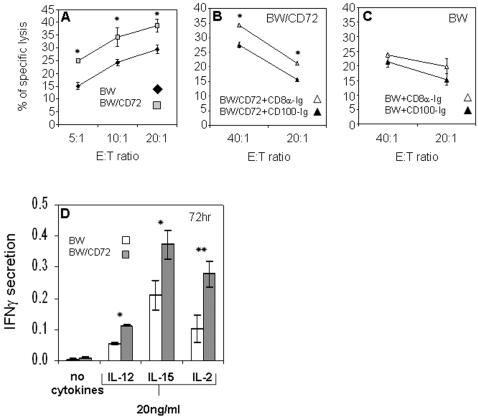
NK cytotoxicity and IFNγ secretion is enhanced after binding to CD72. (A) ^35^S-labeled BW or BW/CD72 cells were tested for lysis by activated NK cells at different effectors to targets ratio as indicated in the figure. *p<0.005. (B) ^35^S-labeled BW/CD72 cells were pre-incubated with the indicated fusion proteins and tested for lysis by activated NK cells at the indicated E:T ratios.*p<0.05. (C) ^35^S-labeled BW cells were pre-incubated with the indicated fusion proteins and tested for lysis by activated NK cells at the indicated E:T ratios. The differences observed between the various treatments were not statistically significant. (D) NK cells were incubated with BW or BW/CD72 in the presence or absence of the various cytokines indicated in the figure and IFNγ secretion was measured by ELISA. Figure shows one representative experiment out of four performed. * p = 0.03, **p = 0.01

Human CD100 interacts with CD72, originally identified as a B cell differentiation antigen [22]. To test whether the CD100-CD72 interaction will affect NK cytotoxicity we generated BW cells stably expressing human CD72 (BW/CD72). BW cells were chosen because their lysis by NK cells is relatively low and this allows small differences in killing to be detected. IL-2 activated NK cells killed more efficiently (up to 64% more at E: T ratio of 5∶1) BW cells expressing human CD72 then parental BW (Figure 4A). Blocking of the interaction between CD100 and CD72 by pre-incubating the target cells with CD100-Ig reduced the killing compared with the control fusion protein CD8α-Ig (figure 4B), indicating that the enhanced killing is due to CD100-CD72 interaction. In contrast, pre-incubation of BW cells with either CD100-Ig or CD8α-Ig did not influence the killing of BW cells by NK cells (figure 4C). Thus, CD100 is involved in NK cytotoxicity probably as an adhesion molecule that enhancing the killing of different target cells expressing CD72.

To test whether CD100-CD72 interactions would modify cytokines secretion, freshly isolated NK cell were incubated with BW or BW/CD72 cells in the absence or presence of NK activating cytokines for 72 hr (figure 4D). Fresh NK cells incubated with BW or BW/CD72 cells did not secrete IFNγ (figure 4D). However, when the (NK) activating cytokines were added to the assay, NK cells that were incubated with BW/CD72 secreted significantly higher amount of IFNγ as compare to NK cells incubated with BW cells. This phenomenon was restricted to IFNγ, because no differences in the secretion of TNFα or GM-CSF were observed when NK cells were incubated either with BW or BW/CD72 (data not shown).

### CD100 on NK cells is an adhesion protein

**Figure 5:**
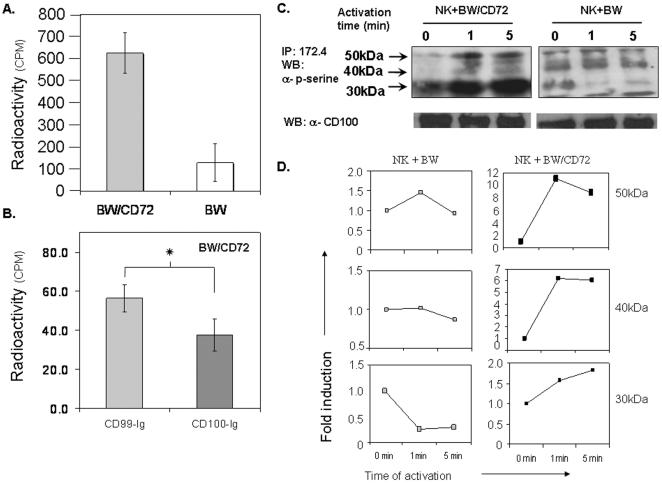
The interaction between CD100 and CD72 leads to the phosphorylation of serine residues on proteins associate with CD100. (A) ^35^S-labeled activated NK cells were incubated for 20 minutes with adherent BW or BW/CD72 cells. The wells were washed, the remaining cells were lysed and the level of radioactivity was measured in CPM units (counts per mint). The results presented after subtraction of NK cells only. (B) ^35^S-labeled activated NK cells were incubated for 20 minutes with adherent BW/CD72 cells that were pre incubated with CD100-Ig or CD99-Ig for 2 hr prior to the incubation with NK cells. The wells were washed, the remaining cells were lysed and the level of radioactivity was measured. *p = 0.02. (C) Activated NK cells were incubated with target cells (BW or BW/CD72), in 37°C, for the indicated time periods. Cells were lysed and proteins were immunoprecipitated by mAb 172.4. The immunoprecipitated proteins were separated on a reducing SDS-PAGE. Phosphorylated proteins were detected in western blot by using rabbit anti phospho-serine polyclonal Ab. CD100 levels were detected in western blot by using the A8 anti human CD100. (D) Densitometrical analysis of the level of phosphorylation on serine residues of the three proteins indicated by arrows in Figure 5C. The levels of phosphorylation are relative to their level at time zero which was set as one. Figure shows one representative experiment out of two performed.

To verify that CD100 function as an adhesion molecule on NK cells we preformed an adhesion assay in which BW or BW/CD72 cells were incubated on 96 flat bottom plate for two hours at 37°C. ^35^S-Met labeled-IL-2-activated NK cells were then added to the plate, washed and radioactivity was measured. As shown in figure 5A, NK cells adhere more strongly to BW cells expressing CD72 compared with BW cells. Blocking CD100-CD72 interaction by pre-incubating BW/CD72 with CD100-Ig reduce the adhesion between the NK cells to the target cells (figure 5B). To further confirm that CD100 is an adhesion molecule on NK cells, we performed signaling experiments. IL-2-activated NK were incubated with BW/CD72 or BW cells for short period of time and then lysed. CD100 was immunoprecipitated with 172.4 mAb. The proteins were eluted under reduce condition and separated on an SDS-PAGE. Western blot analysis with antibody directed against phosphorylated serine residues revealed that cross linking of CD100 by CD72 led to an increase in phosphorylation on serine residues in three unknown proteins that are associated with CD100 (indicated by arrows, figure 5C). In contrast, when NK cells were incubated with BW cells the level of serine phosphorylation did not change or even slightly decreased. To confirm that equal levels of proteins were precipitated in the various treatments, we re-blot the membranes with anti CD100 mAb A8. As shown in figure 5C the level of CD100 is equal in all the lanes. Thus, the changes observed in the levels of serine phosphorylation are genuine. These results were confirmed by the quantification of the level of phosphorylation using densitometrical analysis (figure 5D). Thus CD100 on activated NK cells strengthens their binding to target cells expressing CD72. The interaction with CD72 leads to increase in serine phosphorylation found in unknown proteins associated with CD100.

## Discussion

By immunization of mice with the NK cell line YTS we were able to isolate one mAb named 172.4 that recognized the CD100 protein. The expression of CD100 is up-regulated on the surface of NK cells after their activation with IL-2, IL-12 and IL-15 (figure 1), implicating CD100 as activation marker for NK cells. CD100 can also be used to distinguish between the states of activation of the CD16^+^ and CD16^−^ NK cell populations. Activation of NK cells with IL-12 elevated the level of CD100 on CD16^+^ but not on CD16^−^ NK cells compare with IL-15 which elevated the level of CD100 on both populations (figure 1).

We demonstrated hat CD100 is not a lysis receptor but could instead enhance the killing and cytokine secretion by interacting with its ligand CD72. The interactions between CD100 and CD72 might be important in killing of transformed cells such as leukemia of B cell origin, which express CD72 on their surface [29], [46]–[48].

It has long been established that NK cells can interact directly with dendritic cells [49] and macrophages [50]. It is known that NK cells not only influence DC function via cytokine secretion but also via direct killing [51]. Since CD72 is expressed on antigen presenting cells like B cells, macrophages and dendritic cells, the interaction with NK might also influence the function of these cells via the CD100-CD72 interactions. Of a special note is the possible cross-talk between NK cells and B cells. The fact that that NK express both CD100 and CD40L and the B cells express their counterpart CD72 and CD40 imply that these cells may interact directly. It was demonstrated that soluble CD100 (sCD100) is crucial for the development of Ag-specific immune responses to T cells-dependent (TD) Ag [28], [32], [33]. The immune responses can be restore in these mice by applying sCD100 [28], [32]. But what could be the early source of sCD100? .Both T cell and B cell, express CD100 but they can only release sCD100 after their activation [15], [16], [22], [23] and thus could not be the initial source for sCD100. This raises the possibility that anther cell, probably belonging to the innate system is responsible for early release of sCD100.

We demonstrate here that NK can produce sCD100 form (∼120kDa, figure 3A) and also observed that IL-2-activated NK cells secrete sCD100 (data not shown) and thus we suggest that NK cells are the early source for CD100. We propose the following model. NK cells are activated at the infection site meet APC and secrete sCD100. They interact with APC by several routs among which is the CD100-CD72 interactions. Such interactions lead to IFNγ and sCD100 secretion which in turns activates other cells of the immune system.

## Materials and Methods

### Cells and transfections

The cell lines used were the MHC class I-negative human EBV-transformed B cell line 721.221, simian kidney cells COS-7 and the α/β TCR-negative mouse tymoma BW. Cell lines were grown in complete RPMI-1640 or DMEM (supplemented with 10% FBS, 1 mM L-glutamin, 1 mM penicillin-streptomycin, 1 mM non-essential amino acids and 1 mM sodium pyruvate (Gibco, Minneapolis, MN)). NK cells were isolated from peripheral blood mononuclear cells (PBMC) using the human NK cell isolation kit and an autoMACS instrument (Miltenyi Biotec, Bergisch-Gladbach, Germany.). NK cells were grown as previously described [43]–[45]. BW cell were transfected with pcDNA3.1/V5-His-TOPO (Invitrogen, Carlsbad, CA) contaning the cDNA of human CD72 (BW/CD72). BW/CD72 cells were grown in complete medium RPMI-1640 supplemented with 5 mg/ml G418 (Gibco). The Human CD72 gene was amplified from 721.221 cells using the 5′ primer (with Kozak and Kpn I site): GGTACCCGCCGCCACCATGCTGAGGCCATCACCTATG and the 3′ primer (with Xba I site): GCTCTAGAGCCTAATCTGGAAACCTGAAAGCTAT. The hCD72 was inserted into the plasmid with the TOPO TA expression Kit (Invitrogen).

Monoclonal antibodies used in this work were mAb B73.1.1, directed against CD16 (IgG1), A8 directed against CD100 (Serotec, Oxford, U.K), 3F3 directed against CD72 (Serotec), 12E7 directed against CD99 (IgG1), HC10 directed against the free heavy chain of MHC class I and mAb 172.4 directed against human CD100 (IgG1). For flow cytometry analysis the following Abs were used: PE-conjugated mouse anti-human CD19 (BD PharMingen, San Diego, CA), Cy-Chrome-conjugated mouse anti-human CD3 (BD Pharmingen), FITC-conjugated mouse anti-human CD56 (Serotec), FITC-conjugated mouse anti-human CD16 (Dako, Glostrup, Denmark), 172.4 mAb biotinylated was used as first Ab and then Cy-5 conjugated streptavidin (Jackson Immunoresearch, West Grove, PA).

### Cytotoxicity assays

The cytotoxic activity of NK cells against the various targets was assayed in 5-hr ^35^S-release assays, as described previously [43]–[45]. In all presented cytotoxic assays, the spontaneous release was less than 20% of maximal release. In some assays the NK cells were pre-incubated with 0.5 µg/well of each antibody 1 hr on ice prior to the experiment. In the Re-directed lysis experiment target cells were pre-incubated with increasing concentration of anti-CD16 mAb B73.1.1 with or without 0.1 µg/well of the indicated mAbs. In some assays the target cells were pre-incubated with 40 ng/ml of each fusion protein 2 hours on ice prior to the experiment.

### Radioiodination, immunoprecipitation, and two-dimensional SDS-PAGE

YTS cells were radioiodinated with 1 mCi of Na^125^I (ICN Biomedicals, Inc., Irvine, CA) by the lactoperoxidase method. After extensive washes with cold PBS, labeled cells were solubilized on ice with 0.5% Nonidet P-40/10 mM iodoacetamide/5 mM EDTA/1 mM PMSF/10 µM leupeptin and pepstatin A in PBS, pH 8.3 (lysis buffer). Cell lysates (1×10^6^ cells/sample) were precleared on ice with protein A coupled sepharose beads (Zymed Laboratories Inc, S. San Francisco, CA) precoated with rabbit anti-mouse IgG. Aliquots of precleared lysates were then immunoprecipitated overnight at 4°C with protein A coupled sepharose beads precoated with rabbit anti-mouse IgG and mAb 172.4 or control IgG1 mAb. Immunoprecipitated samples were washed with lysis buffer, and labeled proteins were eluted in the presence of SDS under non-reducing conditions. Non-reduced samples were separated on SDS-PAGE. The gels were then cut into strips, each representing an immunoprecipitate, reduced with 2% (V/V) 2-mercaptoethanol at 37°C for 1 hr, and placed on top of the second dimension SDS-PAGE followed by autoradiography. For detection of phosphorylated proteins, NK cells were first activated by coincubating 20×10^6^ NK cells per sample and 20×10^6^ BW/CD72 cells or BW in 500 µl complete RPMI-1640 medium at 37°C. After activation cells were immediately washed four times with ice cold PBS containing 1∶100 phosphatase inhibitor cocktail II (Sigma-Aldrich, Inc, St Louis, MO). Cells were lysed with 500 µl of lysis buffer (150 mM NaCl/50 mM Tris (pH 7.6)/0.5% Nonidet P-40/5 mM EDTA) supplemented with 1∶100 aprotinin (Sigma-Aldrich)/1 mM PMSF and 1∶100 of phosphatase inhibitor cocktail II (Sigma-Aldrich). Aliquots were precleared on ice with protein A coupled sepharose beads (Zymed Laboratories, Inc.) precoated with irrelevant mouse IgG. Aliquots of precleared lysates were then immunoprecipitated overnight at 4°C with protein A coupled sepharose beads precoated with mAb 172.4. . Immunoprecipitated samples were washed four times with lysis buffer and proteins were eluted in the presence of SDS under reducing conditions. Samples were separated on SDS-PAGE and phosphorylated proteins were visualized by pAbs rabbit anti-phosphoserine (Stressgen, Victoria, BC) and EnVision+ system (Dako). The level of phosphorylation was quantified using the ImageMaster VDS-CL (Amersham Pharmacia biotech, Piscataway, NJ) densitometer. Level of CD100 was detected by using the commercial anti human CD100 antibody A8 (serotec).

### ELISA

96 wells MaxiSorp plates (Nunc, Roskilde, Denmark) were pre-coated overnight at 4°C with increasing concentrations of various fusion proteins in 50 µl of PBS per well. Plates were washed three times with washing buffer (PBS+0.5% Tween 20) and blocked with 200 µl of 1% BSA in PBS for 2 hr on ice. Plates were washed three times with washing buffer and incubated overnight at 4°C with 0.1 µg/well of indicated antibodies in 100 µl of blocking buffer/Tween (PBS+1% BSA+0.05% Tween 20). Plates were washed 4 times with washing buffer and incubated for 1 hr on ice with 100 µl of goat anti-mouse IgG (H+L)-AP conjugated (Bio-Rad, Hercules, CA), diluted 1/2000 in blocking buffer/Tween . Plates were washed six times with washing buffer and incubated for 30–50 min at room temperature with alkaline phosphatase substrate kit (Bio-Rad). Optical density was measured in an ELISA reader at 405 nm.

For the detection of IFNγ secreted by NK cells, 100,000 NK cells per well were incubated with 100,000 BW or BW/CD72 cells in 200 µl of complete RPMI-1640 on flat bottom plate for 72 hours at 37°C with or without 20 ng/ml of the indicated cytokines (Pepro tec.). The ELISA for the detection of human IFNγ was performed in accordance with the manufacture protocol and reagents (PharMingen)

### Adhesion assay

NK cells were label with ^35^S-Met as described previously [43]–[45]. 100,000 BW or BW/CD72 cells per well were incubated in 50 µl complete RPMI-1640 (Gibco) for 2 hr in 37°C on 96 flat bottom plates (Nunc). 50,000 NK cells in 50 µl complete RPMI-1640 medium were added to the plate and incubated for anther 20 minutes at 37°C. The medium was aspirated from the plate gently and then the plate was washed once with 200 µl PBS. 100 µl of RPMI-1640 was added to each well and cells were lysed by adding 100 µl of 0.1 M NaOH. Radioactivity was monitored as describe before [43]–[45]. In some assays the target cells were pre-incubated with 40 ng/ml of CD100-Ig or CD99-Ig, 2 hours in 37°C prior to the experiment.

### Activation of cells with cytokines

PBMC were incubated in 37°C for 72 hr with 50 ng/ml of different cytokines in complete RPMI-1640 medium (Pepro tec., London, U.K).
